# Nasal delivery of nanoliposome-encapsulated ferric ammonium citrate can increase the iron content of rat brain

**DOI:** 10.1186/s12951-017-0277-2

**Published:** 2017-06-02

**Authors:** Xueling Guo, Hong Zheng, Yuetong Guo, Yan Wang, Gregory J. Anderson, Yunzhe Ci, Peng Yu, Lina Geng, Yan-Zhong Chang

**Affiliations:** 1 Key Laboratory of Animal Physiology, Biochemistry and Molecular Biology of Hebei Province, College of Life Sciences, Hebei Normal University, Shijiazhuang, 050024 China; 2 College of Chemistry and Material Science, Hebei Normal University, 20, Nanerhuan Eastern Road, Shijiazhuang, 050024 Hebei China; 3 Iron Metabolism Laboratory, QIMR Berghofer Medical Research Institute, PO Royal Brisbane Hospital, Brisbane, Australia; 4 Laboratory of Molecular Iron Metabolism, College of Life Sciences, Hebei Normal University, 20, Nanerhuan Eastern Road, Shijiazhuang, 050024 Hebei China

**Keywords:** Nasal delivery, FAC, Nanoliposomes, ICP-MS, Micro-X-ray fluorescence

## Abstract

**Background:**

Iron deficiency in children can have significant neurological consequences, and iron supplementation is an effective treatment of choice. However, traditional routes of iron supplementation do not allow efficient iron delivery to the brain due to the presence of the blood–brain barrier. So an easily delivered iron formulation with high absorption efficiency potentially could find widespread application in iron deficient infants.

**Results:**

In this study, we have developed and characterized a nanovesicular formulation of ferric ammonium citrate (ferric ammonium citrate nanoliposomes, FAC-LIP) and have shown that it can increase brain iron levels in rats following nasal administration. FAC was incorporated into liposomes with high efficiency (97%) and the liposomes were small (40 nm) and stable. Following intranasal delivery in rats, FAC-LIP significantly increased the iron content in the olfactory bulb, cerebral cortex, striatum, cerebellum and hippocampus, and was more efficient at doing so than FAC alone. No signs of apoptosis or abnormal cell morphology were observed in the brain following FAC-LIP administration, and there were no significant changes in the levels of SOD and MDA, except in the cerebellum and hippocampus. No obvious morphological changes were observed in lung epithelial cells or tracheal mucosa after nasal delivery, suggesting that the formulation was not overtly toxic.

**Conclusions:**

In this study, nanoscale FAC-LIP proved an effective system delivering iron to the brain, with high encapsulation efficiency and low toxicity in rats. Our studies provide the foundation for more detailed investigations into the applications of niosomal nasal delivery of liposomal formulations of iron as a simple and safe therapy for iron deficiency anemia.

**Graphical abstract:**
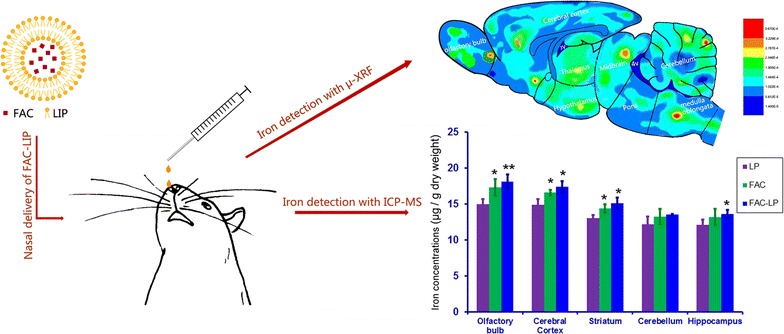
The diagrammatic sketch of “Nasal delivery of nanoliposome-encapsulated ferric ammonium citrate can increase the iron content of rat brain”. Nanoliposome-encapsulated ferric ammonium citrate (FAC-LIP) was successfully prepared and intranasal administration of FAC-LIP increased both the total iron contents and iron storage protein (FTL) expression in rat olfactory bulb, cerebral cortex, striatum and hippocampus, compared with those of FAC groups. Moreover, there was not overtly toxic affects to brain, lung epithelial cells and tracheal mucosa.

**Electronic supplementary material:**

The online version of this article (doi:10.1186/s12951-017-0277-2) contains supplementary material, which is available to authorized users.

## Background

Iron is absolutely required for virtually every cell. In mammals, iron is particularly important for oxygen transport, DNA synthesis, neurotransmitter synthesis and as an electron acceptor/donor in energy transduction [1, 2]. Iron deficiency is the most common type of micronutrient deficiency worldwide and is listed as one of the World Health Organization’s top 10 target diseases for cure and prevention [3, 4]. Maternal iron deficiency during gestation and breastfeeding has been associated with serious and irreversible neurological impairment in the infant, including motor, social, emotional, neurophysiological and neurocognitive dysfunction [5, 6]. Iron supplementation is an effective method for treating iron deficiency. Although traditional iron supplements such as ferrous sulfate, ferrous gluconate and ferrous fumarate are commonly used in clinical practice to treat iron deficiency-related conditions [3, 7], their efficiency in crossing the blood–brain barrier (BBB) is low. Thus, new supplements which enter the brain more readily are needed.

Liposomes are small vesicles enclosed by a lipid bilayer membrane. They are useful as efficient delivery vehicles for drugs, plasmids, peptides, proteins, viruses and bacteria as they have high solubility and bioavailability, and have low toxicity and are non-immunogenic [8, 9]. Our previous work has shown that iron encapsulated in liposomes can be absorbed effectively by intragastric administration [10], and nanoliposomes have been predicted to be even more efficient for carrying cargo across the BBB due to their optimized size, shape and surface coating [9, 11]. Ferric ammonium citrate (FAC) is a water soluble iron chelate that has been used as an iron supplement [12]. However, FAC does not efficiently enter the brain due to the regulation of the iron uptake proteins in BBB [13]. Encapsulating it in nanoliposomes has the potential to allow the direct delivery of FAC to the brain, thereby bypassing the physiological iron uptake mechanism. The route of delivery is also important, as it is often difficult to administer drugs to infants, so the nasal route has considerable appeal. Intranasal delivery can be carried out easily. It is a noninvasive method that can bypass the BBB and is ideal for targeting iron nanoliposomes to the brain [14]. The advantage of delivering drugs to the brain via the nasal mucosa is that it provides a rapid access route and therefore correspondingly rapid therapeutic effects [15, 16]. Thus, administering nanoliposomes by niosomal nasal delivery has considerable potential for targeting drugs across the BBB in children.

In this study, we prepared and characterized nanoliposome-encapsulated FAC and showed that this formulation was able to deliver iron efficiently to the rat brain via the intranasal route.

## Methods

### Materials

Ferric ammonium citrate and a rabbit antibody against β-actin were obtained from Sigma-Aldrich (USA). Soya bean lecithin (a source of phospholipids) was purchased from Beijing Huaqing Meiheng Biotechnology Co. Ltd. (China). Cholesterol was purchased from Beijing Dingguo Biotechnology Co. Ltd. (China). A rabbit polyclonal antibody against L-ferritin was obtained from Abcam (United Kingdom), and rabbit monoclonal antibodies against B cell lymphoma 2 (Bcl-2) and Bcl-2-associated X protein (Bax) were purchased from Santa Cruz Biotechnology (USA). The terminal deoxynucleotidyl transferase-mediated FITC-dUDP nick-end labeling (TUNEL) kit was from TaKaRa (Japan). All other analytical reagents and chemicals were purchased from Bio-High Technology Development Co. Ltd. (China).

### Preparation of FAC nanoliposomes

Ferric ammonium citrate nanoliposomes (FAC-LIP) were prepared by the ethanol injection method [17]. Cholesterol and lecithin were dissolved in ethanol and added drop by drop into a PBS (pH 8.0) solution containing FAC at 50 °C with constant stirring, and then a suspension was formed after evaporation of the ethanol over a period of 30 min. The suspension was dispersed by ultrasonication for 10 min (100 W), dialyzed (MWCO 3500) for 4–5 h, then passed through a 0.22 µm filter. The resulting FAC-LIP nanoparticles were stored protected from light and under N_2_ at 4 °C. Empty liposomes (LIP) were prepared according to the method described above without adding FAC.

### Determination of encapsulation efficiency by ultraviolet spectrophotometry

The amount of FAC encapsulated in the liposomes was determined as previously described [18]. Essentially, FAC-LIP was dialyzed to remove free FAC. The residual sample was placed in a 10 mL centrifuge tube, and an equivalent volume of xylene was added to demulsify the liposomes. Subsequently, the tube was centrifuged at 3500×*g* for 20 min and then at 4000×*g* for 10 min at 25 °C [19]. To measure the concentration of packaged FAC, a standard curve was generated by measuring the absorption at 260 nm of standard FAC solutions (0.0125–0.25 mg/mL). The A_260_ of the encapsulated FAC was measured and the concentration read off the standard curve. The encapsulation efficiency of FAC-LIP was calculated using the following equation: ${\text{Encapsulation efficiency }}\left( \% \right) = \left( {\text{amount of FAC in liposome}} \right)/\left( {\text{total FAC in the system}} \right) \times 100$ [17].

### Measurements of particle size and zeta potential

To determine the diameter of FAC-LIP and empty liposomes, they were diluted 10 times with water and measured using a ZetaSizer Nano series Nano-ZS (Malvern Instruments Ltd, Malvern, UK). All measurements were carried out at room temperature.

### Morphological observations of FAC nanoliposomes by transmission electron microscopy

Ferric ammonium citrate nanoliposomes and LIP were observed by transmission electron microscopy (TEM). Five microliters of liposomes were dropped gently onto amorphous carbon-coated copper grids, and then 10 μL of a uranyl acetate solution was added onto the grids for 10 min. The excess solution was removed with filter paper. Images were captured using a JEM100SX transmission electron microscope (Hitachi, Japan).

### Animals and treatments

Male Sprague–Dawley (SD) rats, approximately 200 g, purchased from HEB LAC (Hebei Medical University, China), were housed in cages at 21 ± 2 °C and provided free access to food and water. Rooms were humidity controlled under a cycle of 12 h of light and 12 h of darkness. All rats were allowed to adapt to their living conditions for 3 days before experiments.

For niosomal nasal FAC administration, conscious rats were grasped gently, and laid on the sloping cage cover, then the head of the rat was elevated to keep the nasal cavity upward. FAC liposomes, FAC or empty liposomes were administered dropwise to the nostrils using a blunt needle. Droplets (a total of approximately 26 droplets) were administered alternately to the left and right nostrils at intervals of 5 s. After the final droplet, the rat’s nose was kept pointing upward for a further 20 s. Each rat received two rounds of nasal drug administration per day (in the morning and afternoon). On each occasion, the FAC-LIP group received 45 μL of FAC-LIP (5.27 μg FAC per μL), the FAC group 45 μL of FAC (5.27 μg/μL), and the LIP group 45 μL of empty liposomes. FAC-LIP and control formulations were delivered for 7 days, then rats were culled and tissues were collected for analysis at 24 h after the last administration. All procedures were carried out in accordance with the National Institutes of Health Guide for the Care and Use of Laboratory Animals and were approved by the Animal Ethical and Welfare Committee of Hebei Normal University (approval no. IACUC-137006).

### Preparation of histological sections

Rats were anesthetized with sodium pentobarbital (40 mg/kg, i.p.) 24 h after the final administration of nanoliposomes. The animals were perfused through the ascending aorta with ice-cold normal saline (0.9% NaCl) followed by 4% paraformaldehyde in 0.1 M phosphate-buffer (PB, pH 7.2–7.4) [20]. The brain, trachea and lungs were removed, post-fixed with 4% paraformaldehyde for another 1.5–4 h, and then stored overnight in 30% sucrose. Sagittal sections of the brain (20 µm thick) were cut using a freezing microtome and fixed onto 3 mm thick Mylar films (polycarbonate) for the analysis of iron distribution by synchrotron-based micro-X-ray fluorescence. Coronary sections of the brain (20 µm thick) were cut using a freezing microtome and mounted onto slides covered with APES (Beijing Zhongshan Biotechnology, Beijing, China) for Nissl staining and the assessment of apoptosis using the TUNEL assay [21]. Sections (6 µm) of the trachea and lung were stained with hematoxylin and eosin (H&E) as previously described [22].

### Determination of iron distribution in brain regions by using synchrotron-based X-ray fluorescence

Micro-X-ray fluorescence (μ-XRF) microspectroscopy was performed soon after the preparation of histological sections. μ-XRF was conducted at the 4W1B endstation of the Beijing synchrotron Radiation Facility, which runs a 2.5 GeV electron beam with current from 150 to 250 mA. The incident X-ray energy was monochromatized by a W/B_4_C Double-Multilayer-Monochromator (DMM) at 15 keV and focused down to 50 μm in diameter with a polycapillary lens. Two-dimensional maps were acquired by step mode: the sample was held on a precision motor-driven stage, scanning 500 μm stepwise. A Si(Li) solid state detector was used to detect X-ray fluorescence emission lines with a live time of 12 s. Data reduction and processing were performed using the PyMCA package [23].

### Inductively coupled plasma mass spectroscopy

The total iron content in brain tissue was determined using inductively coupled plasma mass spectroscopy (ICP-MS) (Thermo Fisher, X Series, FL, USA). Before the experiment, all tubes were soaked in 2 M nitric acid for 24 h, washed with deionized water and then rinsed with ultra-pure water before drying. Approximately 20 mg of dried brain tissue was added to 1 mL of ultra-pure nitric acid (69.9–70.0%, J. T. Baker, USA) and 0.15 mL H_2_O_2_ in Teflon digestion tubes, digested in a microwave digestion system for 2 h at 100 °C, and then for 4 h at 200 °C. The totally digested samples were diluted to 5 mL with ultra-pure water. Standard curves ranging from 0 to 200 µg/L were prepared by diluting the iron standard—^57^Fe (NO_3_) (1 mg/mL). Standards and digested samples were read in triplicate by ICP-MS [24].

### Western blotting

The expression of ferritin (FTL), Bcl-2 and Bax was assessed by Western blotting as previously described [21, 25]. The blots were incubated with FTL antibody (1:5000), Bcl-2 antibody (1:300) and Bax antibody (1:1000) overnight with constant agitation at 4 °C, followed by incubation with an anti-rabbit secondary antibody (Amersham, UK) conjugated to horseradish peroxidase (1:5000) for 1 h at room temperature. Finally, the immunoreactive proteins were detected using chemiluminescence (ECL kit, Pierce, USA), and signals were quantitated with a Fujifilm Luminescent Image Analyzer LAS4000. All experiments were performed at least three times, and the intensities of specific bands were determined and analyzed with NIH imaging software. To ensure even loading of the samples, the same membrane was immunostained with a β-actin antibody (1:5000). All raw values were standardized to the β-actin endogenous control.

### Apoptosis detection by TUNEL assay

Cell apoptosis was assessed in the olfactory bulb, cerebral cortex, striatum, cerebellum and hippocampus by the TUNEL method according to the manufacturer’s instructions, as described previously [21, 26]. Nuclei were stained with DAPI.

### Measurement of superoxide dismutase and lipid peroxidation levels

Superoxide dismutase (SOD) and malonyl dialdehyde (MDA) were used as indicators of oxidative damage as previously described [10, 21]. SOD and MDA were measured using commercial kits according to the manufacturer’s instructions (Nanjing Jiancheng Bioengineering Institute, Nanjing, China). The levels of SOD and MDA were determined in the olfactory bulb, cerebral cortex, striatum, cerebellum and hippocampus after nasal delivery of LIP, FAC or FAC-LIP. Each group contained five mice.

### Quantitative real-time PCR

Total RNA was extracted from the lung 24 h after final liposomes using TRIzol reagent (invitrogen, USA) according to the manufacturer’s instructions. Total RNA was reverse transcribed and real-time PCR was performed as previously described [27]. The primers used for the detection of inflammation are as follows: IL-6 (forward primer: 5′-TCCTACCCCAACTTCCAATGCTC-3′, reverse primer: 5′-TTGGATGGTCTTGGTCCTTAGCC-3′), TNF-α (forward primer: 5′-AAATGGGCTCCCTCTCATCAGTTC-3′, reverse primer: 5′-TCTGCTTGGTGGTTTGCTACGAC-3′) and IL-1α (forward primer: 5′-AAGACAAGCCTGTGTTGCTGAAGG-3′, reverse primer: 5′-TCCCAGAAGAAAATGAGGTCGGTC-3′) [28]. Expression of IL-6, TNF-α, and IL-1α mRNA were normalized to the respective β-Actin levels (forward primer: 5′-AAGTCCCTCACCCTCCCAAAAG-3′, reverse primer: 5′-AAGCAATGCTGTCACCTTCC C-3′).

### Statistical analysis

All experiments were performed at least in triplicate, and the number of animals in each group is provided in the figure legends. Differences between means were determined using a one-way analysis of variance (ANOVA) followed by post hoc multiple comparisons. Differences were considered significant for P < 0.05. All the tests were performed with SPSS 21.0.

## Results

### Characterization of FAC nanoliposomes

Encapsulation efficiency is a critical parameter that must be optimized in the development of a delivery system [18]. An FAC standard curve was prepared in Britton–Robinson buffer (pH  2.0) and used for the determination of encapsulation efficiency. Relative to the starting solution, 97% of the FAC was incorporated into nanoliposomes and the concentration of FAC was 5.27 μg/μL in FAC nanoliposomes.

**Fig. 1:**
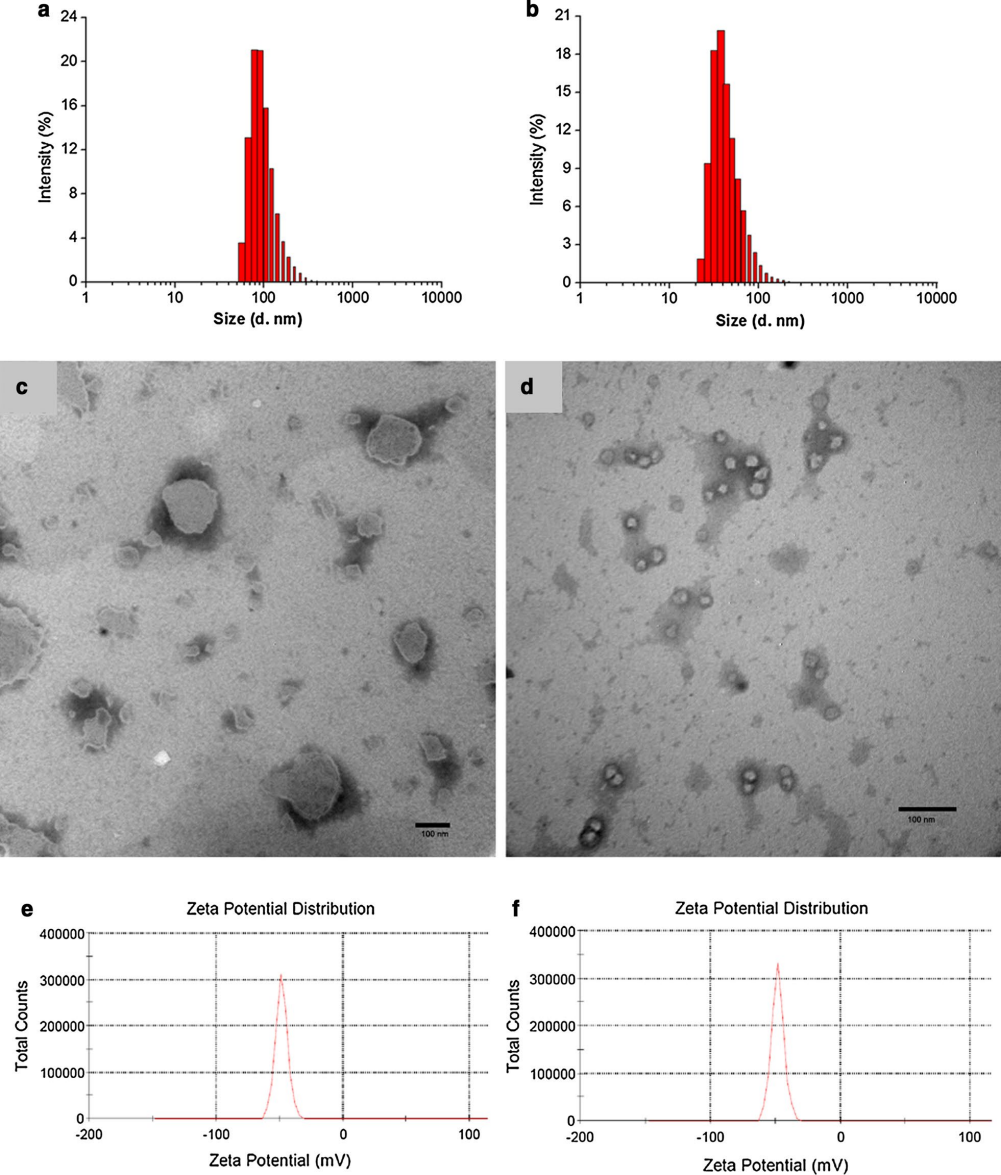
Physical characterization of nanolipsosomes. **a** Diameter of LIP by DLS. **b** Diameter of FAC-LIP by DLS. **c** Morphology of LIP by TEM. **d** Morphology of FAC-LIP by TEM. **e** Zeta-potential of LIP. **f** Zeta-potential of FAC-LIP. *Scale bars* 100 nm

Dynamic light scatting (DLS) indicated that the average hydrated diameters of LIP (Fig. 1a) and FAC-LIP (Fig. 1b) were approximately 80 and 40 nm, respectively. TEM showed that LIP (Fig. 1c) and FAC-LIP (Fig. 1d) were irregular, but largely spherical in shape with continuous membranes.

The zeta potential is another important characteristic determining liposome stability. Particles with a high zeta potential are self-stabilizing, as their charge inhibits coalescence and enhances stability [29, 30]. In general, liposomes are stable when the absolute value of the zeta potential is greater than 30 mV [31]. In our study, the zeta potentials of empty liposomes and FAC nanoliposomes were determined to be −48.3 and −48.1 mV (Fig. 1e, f), respectively, reflecting the high physical stability of the liposomes.

### Elemental mapping of iron in different brain regions after intranasal administration of iron nanoliposomes

**Fig. 2:**
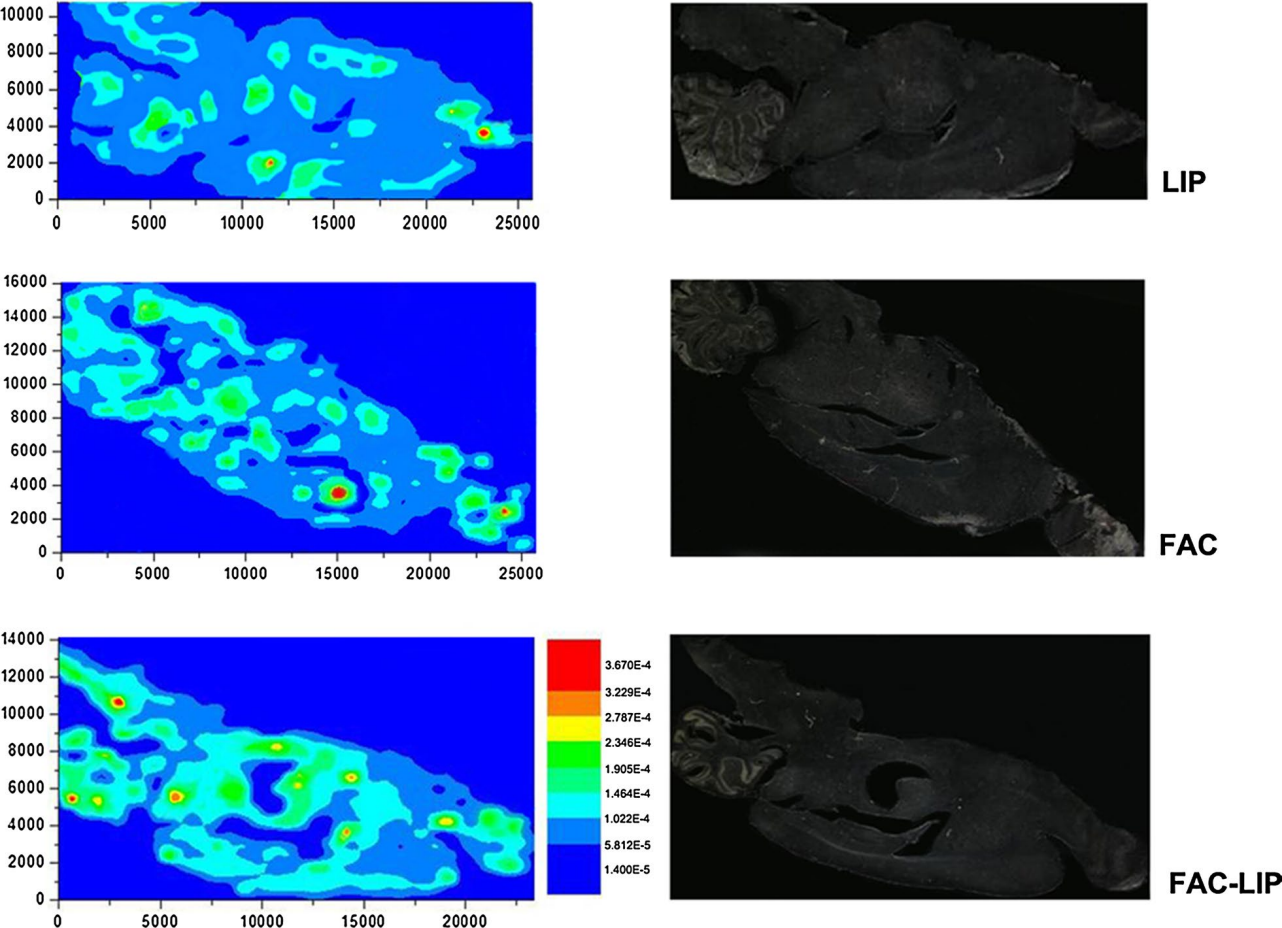
Distribution of iron in rat brain after intranasal administration of LIP, FAC or FAC-LIP. Sagittal brain sections were cut as described in “Methods”. The *left* three images show the mapping results with μ-XRF, and iron levels in different brain regions are indicated by *different colors*. *Blue* indicates the lowest iron level, and *red* indicates the highest iron level as shown in the illustration. The *right* three images show the morphology of the brain sections corresponding to the *left* images. n = 3

Micro-X-ray fluorescence is a powerful tool for morphological mapping of trace element distributions. It can allow direct visualization of the metal ion distribution in tissues or cells [23]. μ-XRF analysis showed that iron levels varied between different brain regions, and that overall, the iron content increased in the rat brain after intranasal administration of FAC and FAC-LIP (Fig. 2). Iron levels were significantly elevated in the olfactory bulb, which is a point of entry of drugs into the brain following intranasal administration. High levels of iron also appeared in the striatum, cerebral cortex, cerebellum and hippocampus after FAC-LIP supplementation, indicating that FAC-LIP was absorbed efficiently via the intranasal route.

### Increased brain iron after intranasal administration of iron nanoliposomes

**Fig. 3:**
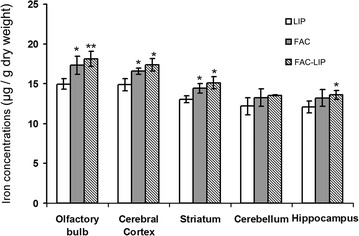
Total iron content in rat brain after transnasal delivery of LIP, FAC and FAC-LIP. The olfactory bulb, cerebral cortex, striatum, cerebellum and hippocampus were dissected, collected and measured with ICP-MS as described in “Methods”. Data are expressed as the mean ± SD. *P < 0.05 and **P < 0.01 vs. LIP group. n = 6

**Fig. 4:**
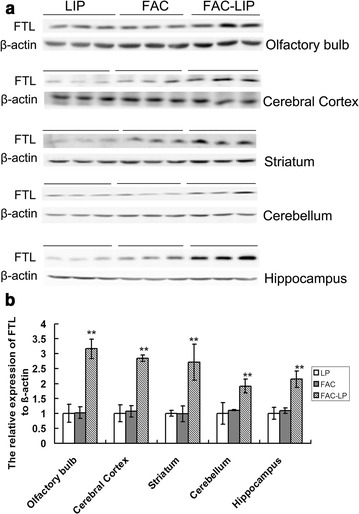
FTL protein expression in rat brain after intranasal administration of LIP, FAC and FAC-LIP. The olfactory bulb, cerebral cortex, striatum, cerebellum and hippocampus were dissected and the expression of FTL protein was detected with western blotting as described in “Methods”. **a** Shows the immunostained bands for FTL (21kD) and β-actin (42 kD), the density of the bands was analyzed and the statistical data was presented in **b**. Relative expression levels were normalized by β-actin and expressed as the mean ± SD. **P < 0.01 vs. LIP group. n = 6

ICP-MS analysis showed that iron levels were significantly increased in the olfactory bulb, striatum, cerebral cortex and hippocampus after nasal delivery of FAC-LIP, compared with FAC treatment alone (Fig. 3). The expression of FTL protein, which correlates positively with intracellular iron levels, was also significantly increased in the olfactory bulb, striatum, cerebral cortex, cerebellum and hippocampus of FAC-LIP treated rats, compared to that of the LIP and FAC treatment groups (Fig. 4).

### Transnasal delivery of FAC-LIP does not induce cell apoptosis in the brain

**Fig. 5:**
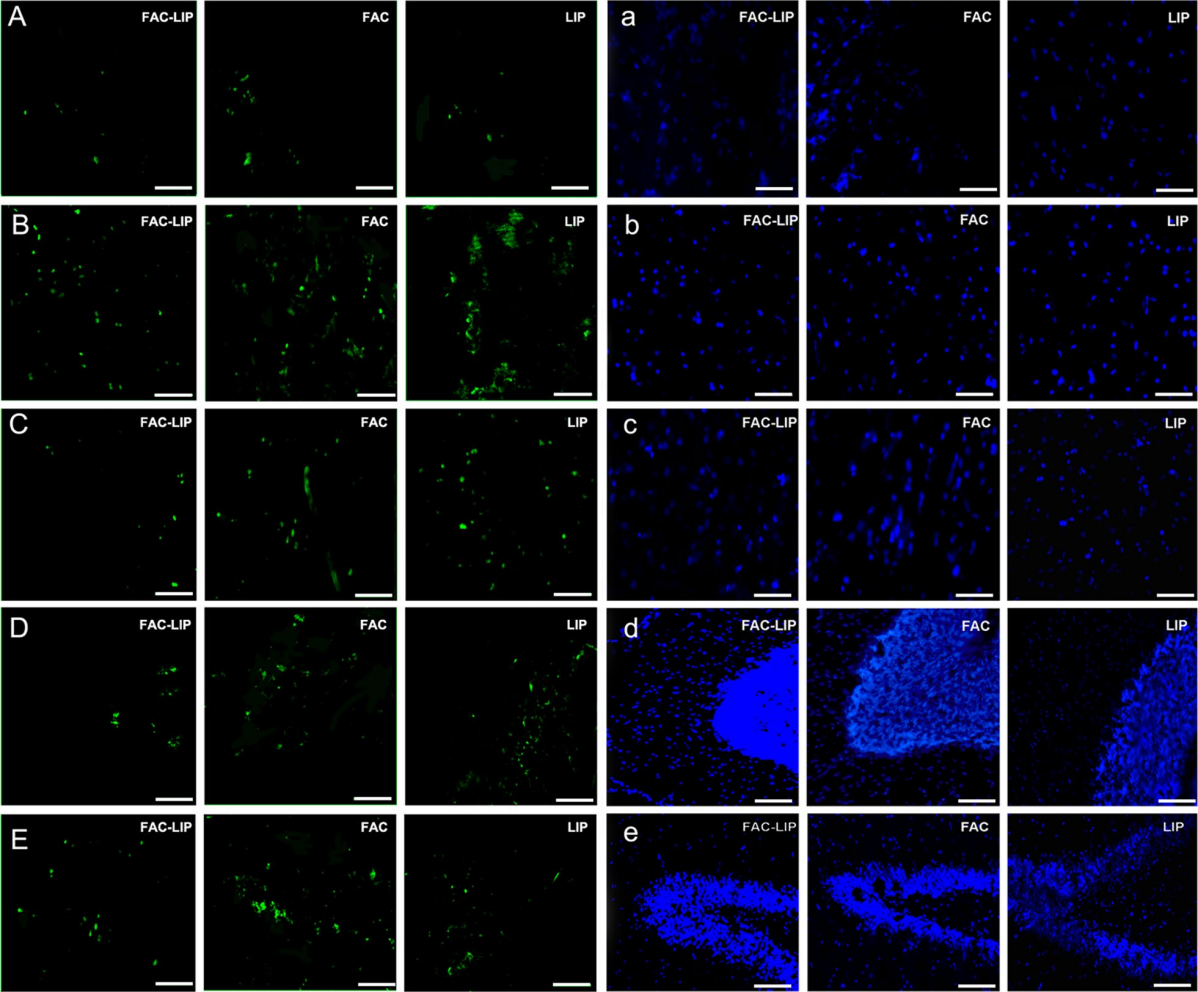
Cell apoptosis was assessed by TUNEL staining. The nuclei were stained with DAPI. In the *left panel*,* green* fluorescence shows the apoptotic cells in olfactory bulb (**A**), cerebral cortex (**B**), striatum (**C**), cerebellum (**D**) and hippocampus (**E**). In the *right*,* blue* fluorescence shows the cell nuclei corresponding to the *left* sections (**a**–**e**). n = 3

**Fig. 6:**
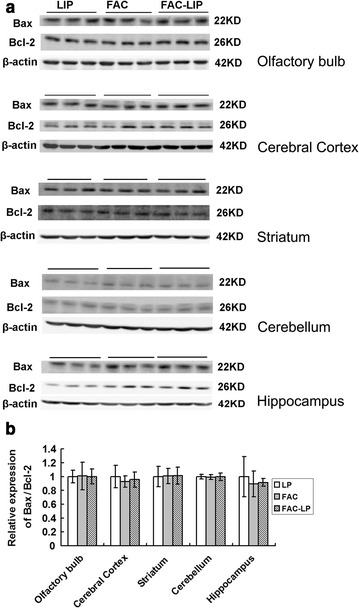
Levels of Bax/Bcl-2 ratio in the brain after transnasal administration of LIP, FAC and FAC-LIP. **a** Western blot analysis of Bax and Bcl-2 protein in olfactory bulb, cerebral cortex, striatum, cerebellum and hippocampus. **b** Histograms of Bax/Bcl-2 ratio of the immunostained bands from **a**. n = 6

Since increased intracellular iron levels can lead to cell apoptosis, the TUNEL assay was used to assess apoptosis in situ in the brain after transnasal delivery of FAC-LIP. No apoptosis was observed in the olfactory bulb, striatum, cerebral cortex, cerebellum or hippocampus, compared to treatment with LIP or FAC alone (Fig. 5). As the Bax/Bcl-2 ratio has been widely used to monitor the degree of apoptosis [21], the expression of the Bcl-2 and Bax proteins was determined by Western blotting following FAC-LIP treatment. Again, no obvious apoptosis was observed following FAC-LIP treatment (Fig. 6).

### Transnasal delivery of FAC-LIP does not induce obvious changes of cell morphology in the brain

**Fig. 7:**
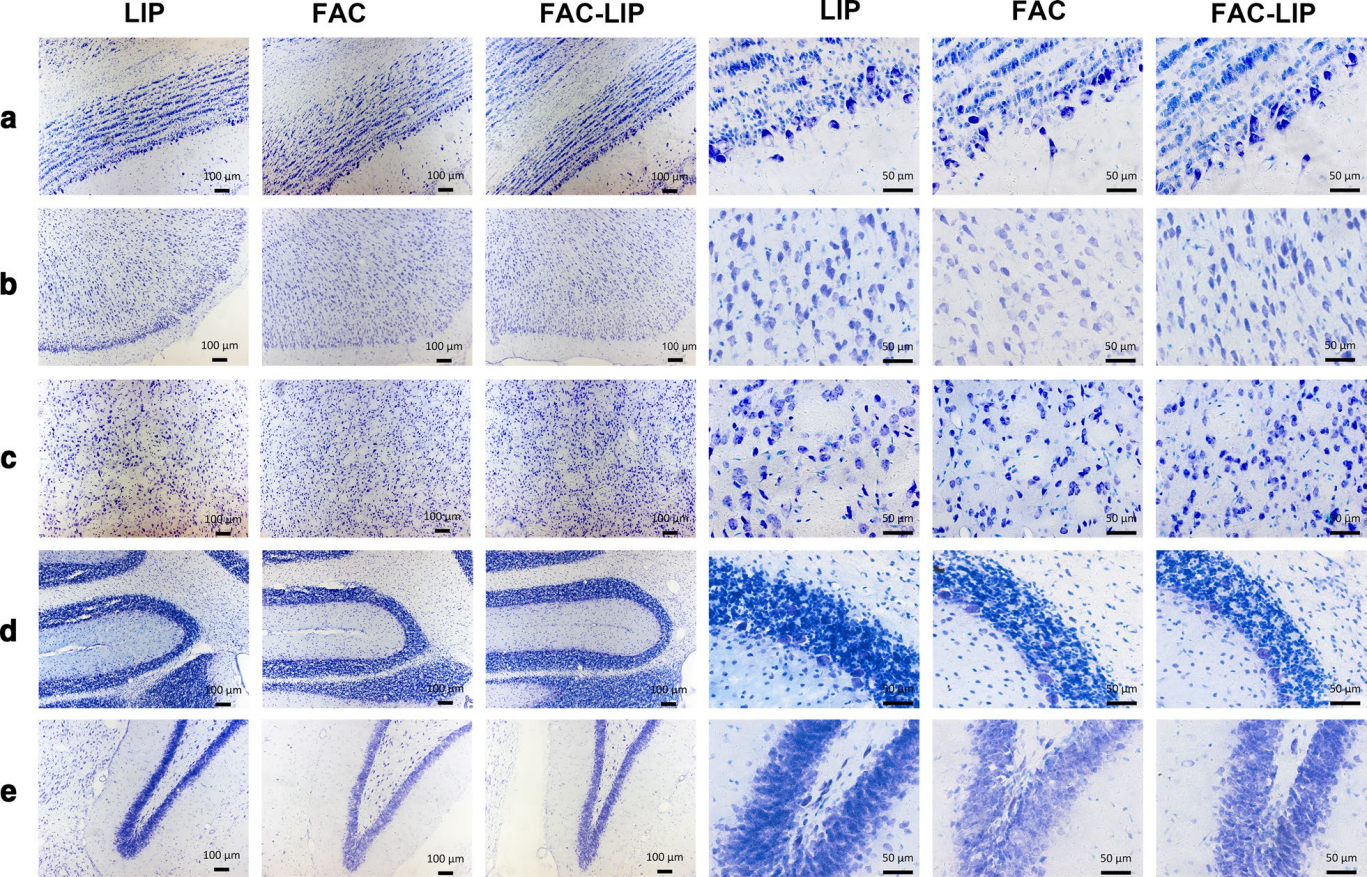
Cell morphology of brain tissues using Nissl staining after transnasal administration of LIP, FAC and FAC-LIP. **a** olfactory bulb, **b** cerebral cortex, **c** striatum, **d** cerebellum, **e** hippocampus, n = 3

Nissl bodies are characteristic of neurons, and Nissl staining is widely used to evaluate the morphology and pathology of neural tissues [32]. In the current studies, Nissl body staining was similar in all treatment groups (Fig. 7) indicating that FAC-LIP did not induce changes of cell morphology in the brain after nasal delivery.

### Levels of SOD and MDA in the brain after transnasal delivery of FAC-LIP

**Fig. 8:**
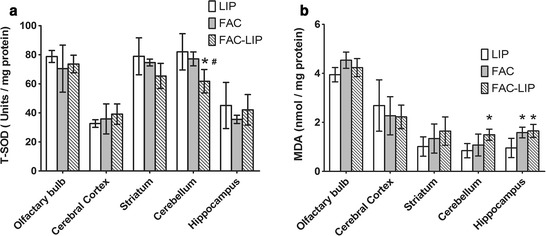
Levels of MDA and SOD in the brain after transnasal administration of LIP, FAC and FAC-LIP. **a** MDA. **b** SOD. Data are presented as mean ± SD, n = 5, *P < 0.05 vs. LIP group, ^#^ *P* < 0.05 vs. FAC group

Superoxide dismutase and MDA are markers of oxidative stress. SODs are enzymes which catalyze the dismutation of superoxide into oxygen and hydrogen peroxide to provide important antioxidant defense in cells. MDA is a reliable marker of lipid peroxidation and measured with thiobarbituric acid. Increased tissue iron levels potentially can induce oxidative damage through the Fenton reaction, so we examined MDA and SOD in the olfactory bulb, striatum, cerebral cortex, cerebellum and hippocampus to evaluate the side effect of nasal delivery of FAC-LIP. FAC-LIP did not induce overt changes in SOD in the olfactory bulb, striatum, cerebral cortex or hippocampus compared with the LIP group, and levels were similar to those of the FAC group (Fig. 8a). Interestingly, however, SOD activity was significantly reduced in the cerebellum relative to LIP and FAC. MDA levels in the FAC-LIP group were not changed in the olfactory bulb, striatum or cerebral cortex compared with the LIP or FAC groups (Fig. 8b). However, MDA levels increased significantly in the cerebellum and hippocampus after nasal administration of FAC and FAC-LIP.

### Transnasal delivery of FAC-LIP did not affect the respiratory system

**Fig. 9:**
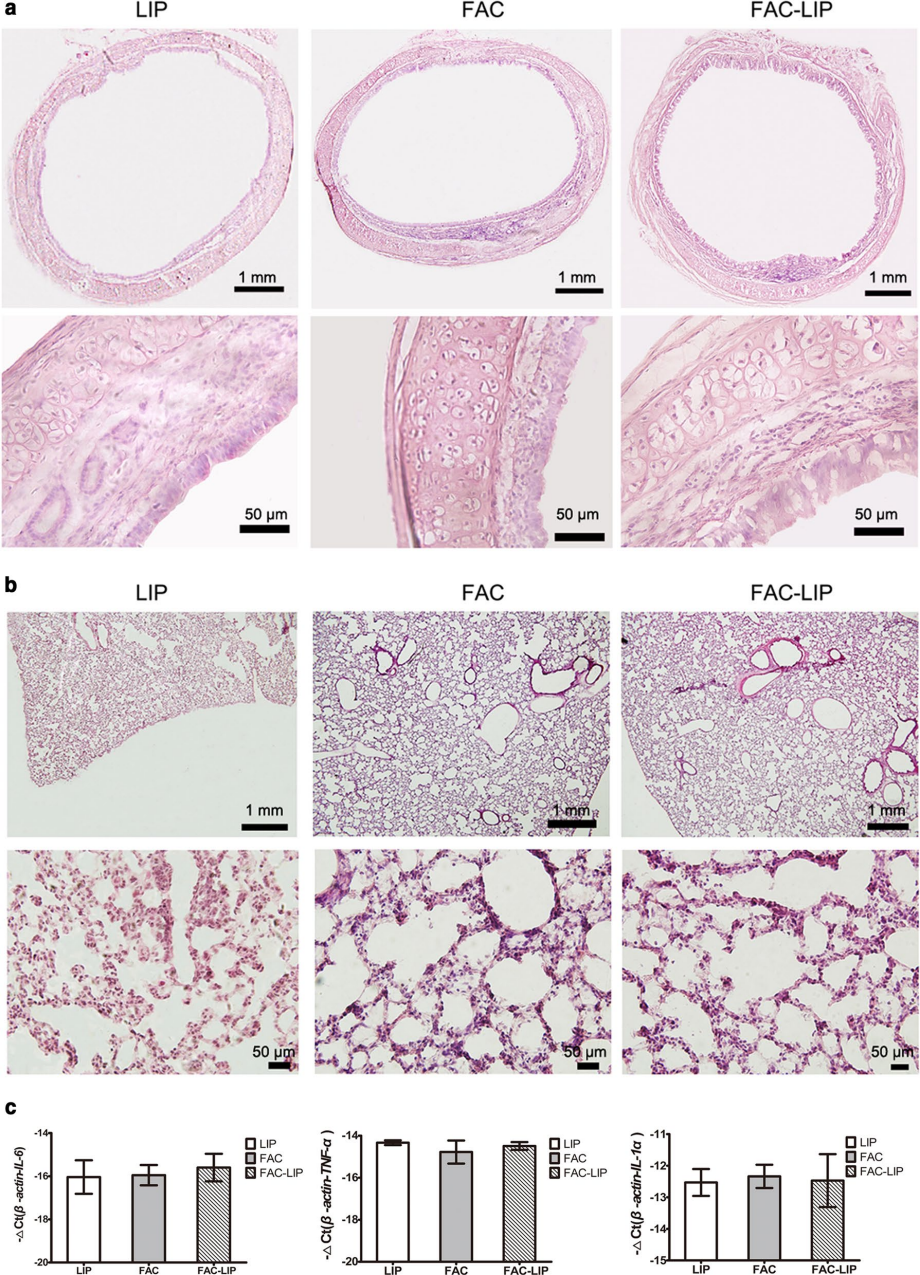
Side effects on trachea and lung tissues after transnasal administration of LIP, FAC and FAC-LIP. **a** Morphology of trachea with H&E staining, n = 3. **b** Morphology of lung with H&E staining, n = 3. **c** Quantitative measurement of IL-6, TNF-α and IL-1α mRNA in lung, results are expressed as −∆Ct ± SD, n = 5

As the iron formulations were administered via the rat nasal passageway and had the potential to pass down into the respiratory tract, toxicity in the trachea and lungs is a potential consequence of treatment. Following FAC-LIP administration, H&E staining showed no obvious changes in the structure of the tunica mucosa in the trachea, and the epithelial mucosa was intact with normal ciliated columnar epithelium cells and goblet cells (Fig. 9a). Alveolar cells in FAC-LIP treated rats were similar to those of LIP treated controls (Fig. 9b). Furthermore, quantitative measurement of IL-6, TNF-α and IL-1α mRNA showed no changes in lung tissues, which showed that no obvious inflammation was induced after nasal delivery of FAC-LIP (Fig. 9c). Thus, no gross adverse consequences were apparent in the respiratory system after transnasal delivery of FAC-LIP to rats.

## Discussion

Iron deficiency can induce anemia and irreversible cognitive dysfunction in the early stages of life. Therefore, giving iron supplementation to children and young infants may be beneficial [33, 34]. Due to the poor absorption efficiency and side effects of traditional iron supplements, we encapsulated FAC into nanoliposomes in order to develop a more efficient iron delivery system. We successfully prepared nanoscale FAC-LIP which were of homogeneous diameter, were physically stable, and had high encapsulation efficiency. The diameter of FAC nanoliposomes was smaller than that of nanoliposomes without cargo. One possible explanation for this relates to the interactions between FAC and the liposomes. We have calculated that the binding Gibbs free energy (∆G) for binding between FAC and LIP in FAC-LIP was −12.7 kJ mol^−1^. This indicates that the interaction is energetically favourable and that FAC can easily be encapsulated in liposomes. Hydrogen bonding and electrostatic interactions between FAC and the liposome membrane could be the driving forces for the encapsulation [34]. In turn, this interaction may cause the liposomes to adopt a more compact formation. Based on the structures of FAC and liposomes, electrostatic interactions may exist between the carboxyl group of FAC and the hydrophilic heads of the phospholipids, while hydrogen bonding could occur between the hydroxyl group of FAC and the methylene group of soya bean lecithin. However, further study is required to define the mechanism in detail. The FAC-LIP nanoparticles were smaller than the FAC liposomes that we previously prepared [10], implying that our newly prepared nano FAC-LIP would be absorbed more efficiently because smaller nanocarriers can cross BBB more easily [11].

Children with iron deficiency face the risk of anemia and neurological disorders [35]. Iron supplements are generally given orally, intravenously or intramuscularly, but these common administration routes are often problematic for children. In particular, conventional iron supplements have a limited ability to cross the BBB, which decreases their efficacy. Thus, a drug delivery system with a higher absorption efficiency than traditional methods that is easy to use could be implemented widely. Intranasal administration is a promising route for delivering drugs effectively to the brain across the BBB [36, 37]. This strategy has been widely applied to deliver therapeutic drugs to the central nervous system as it is associated with fewer systemic effects [16, 38, 39]. Drugs administered intranasally can be transported directly from the nasal mucosa to the brain along the cranial nerves [37]. Therefore, we administered FAC and nano FAC-LIP to rats using the niosomal nasal delivery method. While both free and encapsulated FAC could clearly elevate iron levels in the brain, rats given FAC-LIP showed a significantly higher iron content in the brain than those administered FAC alone. After the absorption of non-heme iron, iron transiently exists in the labile iron pool (LIP) which can meet the demand of cell metabolism [40]. Excess iron is stored in FTL protein, so FAC and FAC-LIP can significantly increase the expression of FTL. However, there are two forms of iron (heme iron and non-heme iron) in the body, and approximately 70% of the total iron exists in the form of heme iron [41]. This is why the increase in total iron content was not as great as the increase in FTL expression.

Intranasal administration is also a promising route of administration due to its higher direct delivery to the brain relative to peroral administration, intraduodenal injection or jugular vein injection [39]. Our data also showed that brain iron content was increased obviously using the transnasal delivery system, and that the delivery efficiency of FAC-LIP is higher than that of FAC, demonstrating that liposome encapsulation is an effective vehicle and intranasal administration is an efficiency and convenient method for iron delivery to the brain. Moreover, investigation of cellular apoptosis in situ, BAX/Bcl-2 moleculars and Nissl staining to examine neuronal cell morphology showed few side effects in the brain of the FAC-LIP treatment. Using SOD and MDA as markers, no obvious oxidative damage was seen in the olfactory bulb, striatum or cerebral cortex. However, the cerebellum and hippocampus did show some increases in oxidative stress in the FAC-LIP group, this may reflect the high efficiency of iron delivery via the nasal route. Determining the optimal dose of iron for delivery through this pathway will require further investigation. Moreover, the chronic toxicity was further assessed at 2 week after nasal administration of FAC-LIP for 7 days, data did not show obvious inflammation in lung (Additional file 1: Figure S1), data did not show obvious oxidative damage (Additional file 2: Figure S2) and cell morphology (Additional file 3: Figure S3) in the brain. The results implied that there was not overtly toxic effect of nasal administration of FAC-LIP on the brain and lung.

In conclusion, a nanoscale FAC-LIP preparation showed favorable characteristics for a drug delivery system, with high encapsulation efficiency and low toxicity in rats. Based on these preliminary findings, niosomal nasal delivery of FAC-LIP offers a potential strategy for developing an effective iron supplement that is capable of delivering iron to the brain efficiently.

## Additional files

**Additional file 1: Figure S1.** Quantitative measurement of IL-6, TNF-α and IL-1α mRNA in lung 2 weeks after transnasal administration of LIP, FAC and FAC-LIP for 7 days. Data are expressed as −ΔCt ± SD, n = 5.

**Additional file 2: Figure S2.** Levels of SOD and MDA in the brain 2 weeks after transnasal administration of LIP, FAC and FAC-LIP for 7 days. Data are presented as mean ± SD, n = 5, * P < 0.05 vs. LIP group, ** P < 0.01 vs. LIP group.

**Additional file 3: Figure S3.** Nissl staining of brain tissues morphology 2 weeks after transnasal administration of LIP, FAC and FAC-LIP for 7 days. A: olfactory bulb, B: cerebral cortex, C: striatum, D: cerebellum, E: hippocampus, n = 3.

## Abbreviations

FAC: ferric ammonium citrate
LIP: liposomes
FAC-LIP: ferric ammonium citrate nanoliposomes
BBB: blood–brain barrier
Bcl-2: B cell lymphoma 2
Bax: Bcl-2-associated X protein
TUNEL: terminal deoxynucleotidyl transferase-mediated FITC-dUDP nick-end labeling
TEM: transmission electron microscopy
PB: phosphate-buffer
μ-XRF: Micro-X-ray fluorescence
FTL: ferritin
DMM: Double-Multilayer-Monochromator
ICP-MS: inductively coupled plasma mass spectroscopy
MDA: malonyl dialdehyde
SOD: superoxide dismutase
DLS: dynamic light scatting
