# Supplementary figures and images for: Nasal delivery of nanoliposome-encapsulated ferric ammonium citrate can increase the iron content of rat brain
Source: J Nanobiotechnology. 2017 Jun 2;15:42. doi: 10.1186/s12951-017-0277-2 (PMC5457662; doi:10.1186/s12951-017-0277-2)

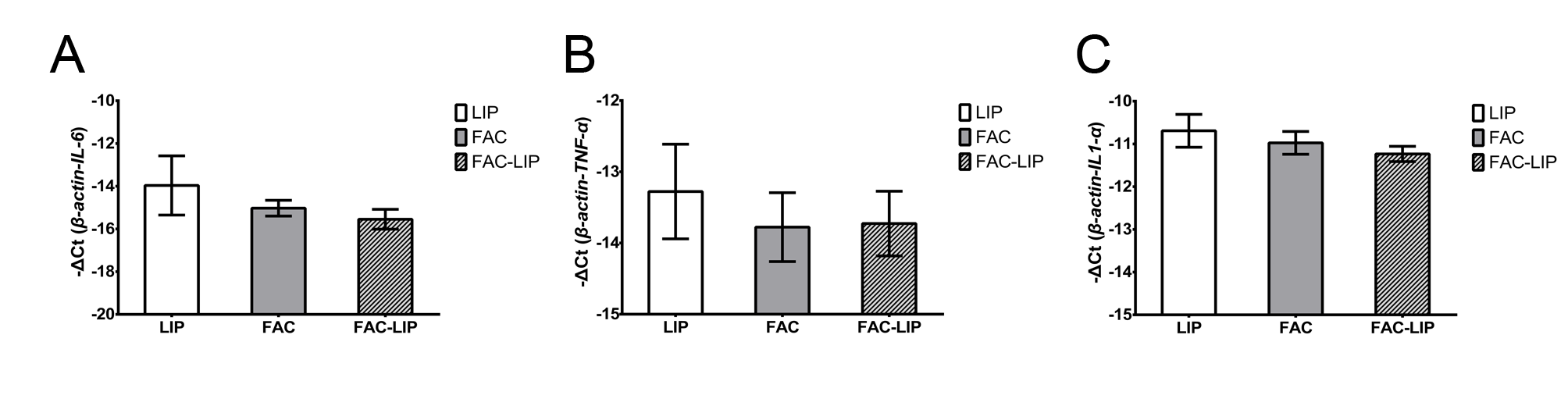

Supplement: Supplementary file 1 — Additional file 1: Figure S1. Quantitative measurement of IL-6, TNF-α and IL-1α mRNA in lung 2 weeks after transnasal administration of LIP, FAC and FAC-LIP for 7 days. Data are expressed as −ΔCt ± SD, n = 5. [file 12951_2017_277_MOESM1_ESM.tif]

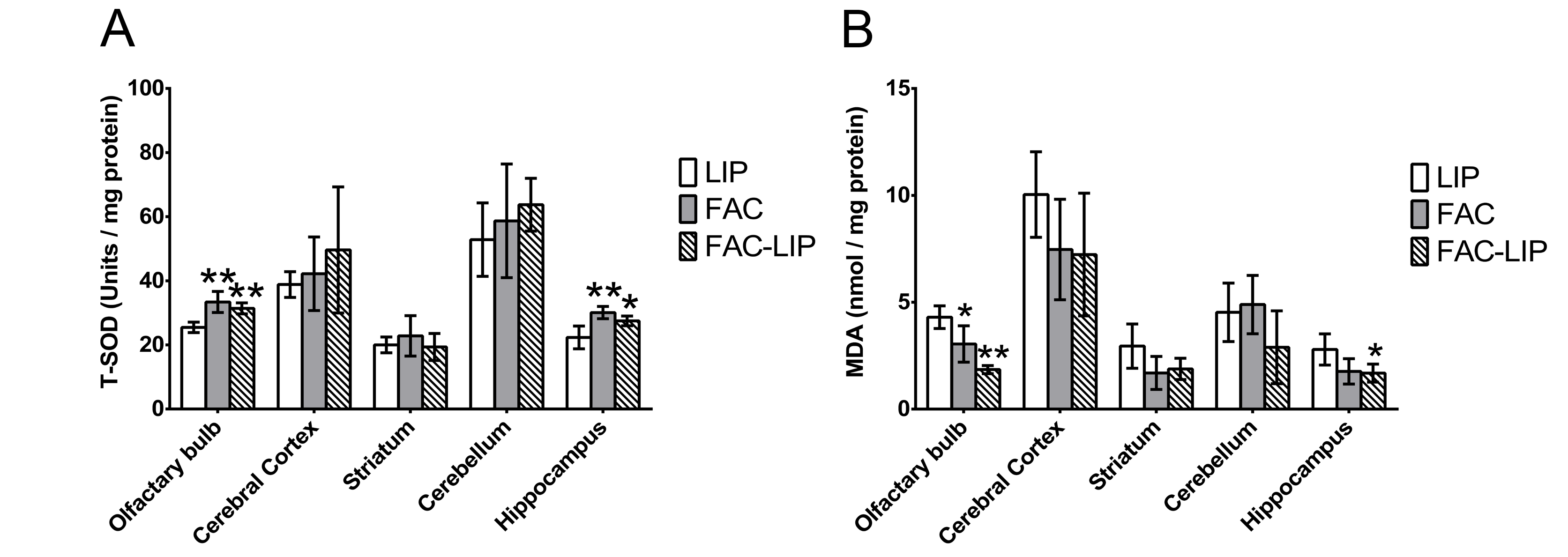

Supplement: Supplementary file 2 — Additional file 2: Figure S2. Levels of SOD and MDA in the brain 2 weeks after transnasal administration of LIP, FAC and FAC-LIP for 7 days. Data are presented as mean ± SD, n = 5, * P < 0.05 vs. LIP group, ** P < 0.01 vs. LIP group. [file 12951_2017_277_MOESM2_ESM.tif]

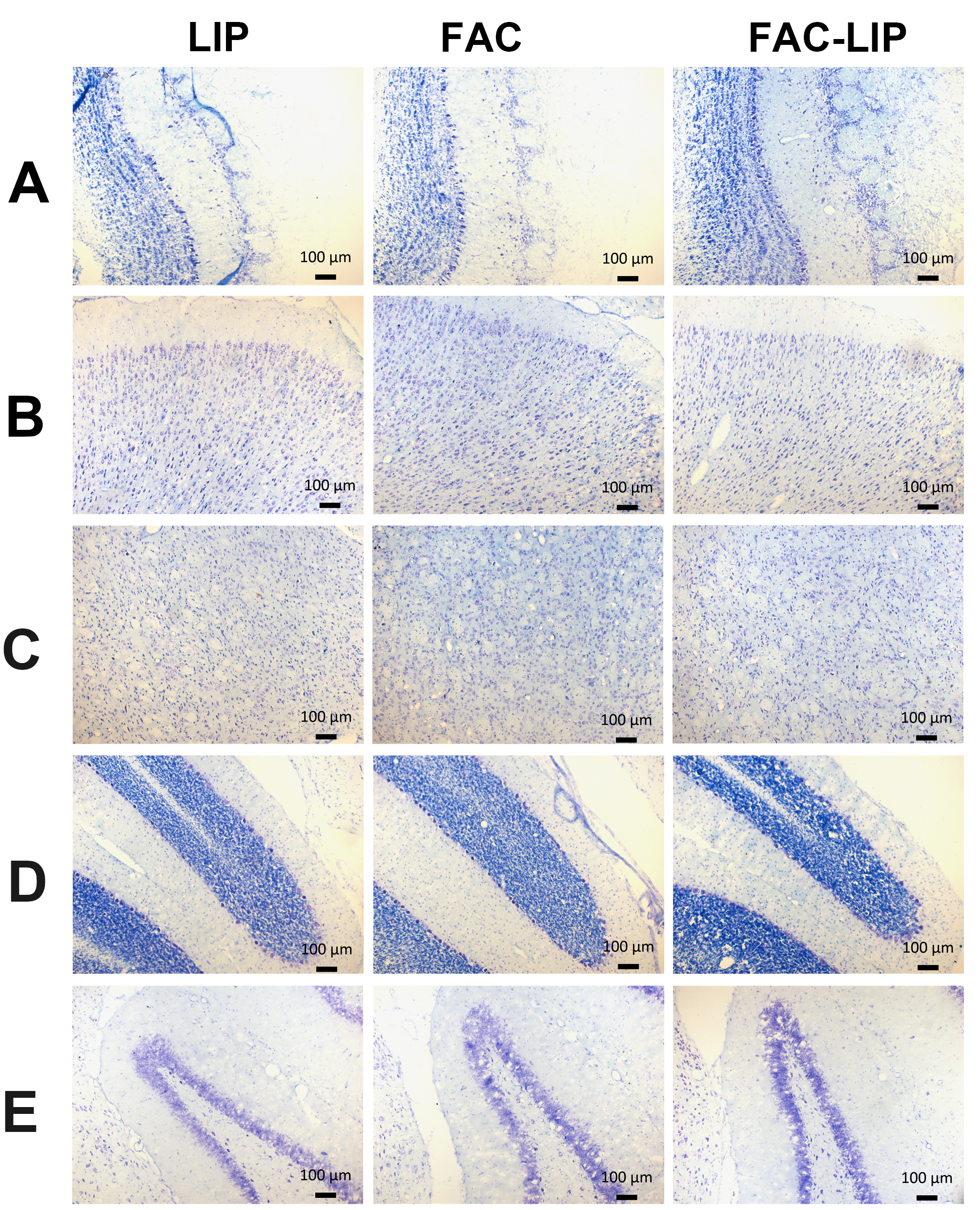

Supplement: Supplementary file 3 — Additional file 3: Figure S3. Nissl staining of brain tissues morphology 2 weeks after transnasal administration of LIP, FAC and FAC-LIP for 7 days. A: olfactory bulb, B: cerebral cortex, C: striatum, D: cerebellum, E: hippocampus, n = 3. [file 12951_2017_277_MOESM3_ESM.tif]
